# Comparison of Amplitude of Accommodation, Near Point of Convergence and Fusion Ability of Islamic Fasters Before, During and After Respected Month of Ramadan

**Published:** 2011-10-01

**Authors:** S H Hoseini Yazdi, E Jafarzadehpur, A Mirzajani, M Nematy

**Affiliations:** 1 Department of Optometry, Faculty of Rehabilitation,Tehran University of Medical Sciences, Tehran, Iran; 2 Department of Nutrition, Faculty ofMedicine, Mashhad University of Medical Sciences, Mashhad, Iran

**Keywords:** Accommodation, Convergence, Fusion, Fasting, Ramadan

Dear Editor,

Fasting during Ramadan month is an Islamic obligation which may have side effects on vision of fasting Muslims. Previous studies showed that there was no relationship between Islamic fasting and myopia[1][2][3][4] and changes in intraocular pressure due to fasting in healthy person were not considerable.[5][6]

In this month, an increased need for visual tasks such as studying Holy Quran, changes in food habits and metabolic conditions necessitate investigations on possible visual changes during this month. Since researches on direct impact of Islamic fasting on vision are few and not conclusive, this study evaluates the amplitude of accommodation (AA), near point of convergence (NPC), positive and negative fusional vergences (PFV and NFV, respectively) of Islamic fasting during Ramadan month.[7]

In our cross sectional study, AA, NPC, PFV and NFV of 30 male students of Tehran University of Medical Sciences were measured during three days before Ramadan month, the middle three days and the three days after this month. Examination time before and after Ramadan was during breakfast and lunch time and from lunch to dinner time while in Ramadan, it was in mid-time between dawn and breakfast meals. To consider nutritional status of subjects over one week before each visit, the Food Frequency Questionnaire (FFQ) was completed.

The mean age and average fasting experience were 23.9 and 10 years, respectively. As seen in Table 1, according to paired t test analysis, AA reduced and NPC increased significantly in Ramadan than before (p<0.01); but there was no significant difference between their values before and after Ramadan. Despite the statistical significance of AA reduction, a change of at least 1.50 D is needed to be considered a significant variation on repeated measurements of accommodative amplitude; smaller changes were accepted as expected variations.[8] On the other hand during Ramadan, the mean AA of the right and left eyes was more than the minimum expected, based on Hoffstetter formula.[9] Thus, despite the reduction of monocular AA during Ramadan, yet its value was within normal range in these young and visually normal subjects. Regarding mean NPC in Ramadan, a range of 8-15 cm was considered normal in some researches.[10][11] As NPC value greater than 10 cm was a diagnostic criteria for convergence insufficiency,[12] it seems that despite the increase in NPC value during Ramadan, this is not clinically significant. Moreover, nutritional pattern analysis showed no significant difference in most nutrient groups before, during and after Ramadan; and significant differences observed in protein and sugar groups had no significant correlation with AA and NPC variations during Ramadan. By reviewing different investigations on Ramadan and Islamic fasting,[13][14][15][16][17] it seems that psychological factors and variations in biological time play a causative role in reduction of AA and increase in NPC in this month.

**Table 1:** Mean±SD of AA, NPC and distant NFV before, during and after Ramadan.

**Variable**	**Before Ramadan**	**During Ramadan**	**After Ramadan**	**P_1_^a^**	**P_2_b**	**P_3_c**
Right Eye Amplitude of Accommodation (Push up method)	13.94±2.67	12.33±2.50	13.37±2.20	<0.0001	0.002	0.184
Left Eye Amplitude of Accommodation (Push up method[7])	13.93±2.68	12.33±2.50	13.33±2.20	<0.0001	0.002	0.086
Near Point of Convergence (Push up method with accommodative target[7])	7.67±3.25	8.70±3.60	7.83±2.76	0.006	0.005	0.267
Negative Fusional Vergence at 6 m, Blur point (Step vergence method[7])	11.24±4.35	8.57±2.23	8.36±2.72	0.003	0.611	<0.0001
Negative Fusional Vergence at 6 m, Break point (Step vergence method[7])	11.21±4.56	8.64±2.37	8.36±2.72	0.005	0.515	<0.0001
Negative Fusional Vergence at 6 m, Recovery point (Step vergence method [7])	8.86±4.23	6.36±2.31	6.29±2.70	0.003	0.865	0.001

^a^ Comparison between before and during Ramadan,

^b^ Comparison between during and after Ramadan,

^c^ Comparison between before and after Ramadan

Although no significant variations were observed in PFV values at far and near and NFV values at near (Repeated measure ANOVA, p>0.05), but NFV values at far significantly reduced during Ramadan than before (Paired t test, p<0.001) and this reduction was not compensated after Ramadan too. As NFV measurements had better repeatability than PFV at both near and distance,[18] therefore the reduction of NFV observed in Ramadan was significant. However, NFV was not needed at far in this sample, as none of the subjects had distance esophoria. Since NFV has a little role in maintaining fusion at distance, it seems that the mechanism to compensate its reduction in Ramadan occurs weakly.

In conclusion, our results showed a considerable increase in NPC value and a significant decrease in AA and distant NFV during Ramadan than before; however the changes were compensated after Ramadan except for NFV at far. Although these changes were not clinically considerable in the young and normal subjects, but in other age groups with larger sample sizes and in individuals with nonstrabismic visual disorders might have clinical impacts. So it is recommended that proper accommodative and convergence trainings are advised by optometrists to Islamic fasters during Ramadan month to prevent these variations. More researches in this field seem necessary.
